# The role of the epithelium in intraocular lens and corneal power calculation

**DOI:** 10.1111/opo.13443

**Published:** 2025-02-03

**Authors:** Jascha A. Wendelstein, David A. Atchison, Damien Gatinel, David L. Cooke, Achim Langenbucher

**Affiliations:** ^1^ University Eye Hospital Ludwig‐Maximilians‐University Munich Germany; ^2^ Institut für Refraktive Und Ophthalmo‐Chirurgie (IROC) Zurich Switzerland; ^3^ Institute of Experimental Ophthalmology Saarland University Homburg Germany; ^4^ Centre for Vision and Eye Research Queensland University of Technology Kelvin Grove Queensland Australia; ^5^ Rothschild Foundation Hospital Anterior Segment and Refractive Surgery Department Paris France; ^6^ Great Lakes Eye Care Saint Joseph Michigan USA; ^7^ Department of Neurology and Ophthalmology, College of Osteopathic Medicine Michigan State University East Lansing Michigan USA

**Keywords:** biometry, corneal power, epithelial curvature, epithelial power, keratometry, stromal curvature, stromal power

## Abstract

**Purpose:**

To investigate the influence of the corneal epithelium on corneal power, particularly in special cases such as post‐refractive surgery and keratoconus.

**Methods:**

A retrospective observational study. Measurement data were obtained from a high‐resolution anterior segment analyser (CSO MS‐39). Corneal curvature and power data, as well as surface height data, were organised in a cylindrical coordinate system. Calculations considered one, two and three refractive surfaces, examining the role of epithelial thickness and stromal curvature.

**Results:**

The effect of the epithelium on corneal power was minimal (<0.1 D) in normal corneas, but it was considerable in keratoconus and post‐refractive surgery cases, with differences up to 0.9 D. The effect decreased for larger measurement zones.

**Conclusion:**

Incorporating epithelial thickness and stromal curvature into corneal power calculations is a crucial next step in accurate corneal power and intraocular lens calculation in eyes with previous refractive surgery or keratoconus. This study highlights the need for advanced diagnostic and calculation methods in complex cases.

**Key points**

- This work evaluates one, two and three refractive surfaces models for corneal power.
- The study provides detailed analysis on the interplay of epithelial and stromal curvature.
- The findings demonstrate significant epithelium impact in eyes with keratoconus or post‐refractive cases and emphasises advanced diagnostics for improved intraocular lens calculations and refractive surgery.

## INTRODUCTION

Recent interest has emerged in corneal power calculations and keratometer indices.^1^, ^2^, ^3^, ^4^, ^5^, ^6^ Traditionally, keratometers and biometers have measured the anterior cornea, incorporating empirical adjustments for corneal thickness and posterior curvature to determine corneal power. With modern tomography and biometry devices, measurements of the posterior cornea and corneal thickness are now available for cataract surgery and intraocular lens (IOL) power calculation.^7^, ^8^ However, the accuracy of IOL power calculation remains limited in cases of special corneas, particularly after refractive surgery such as laser vision correction (LVC) and in conditions like keratoconus (KC), even with the inclusion of posterior corneal measurements.^9^, ^10^, ^11^, ^12^ The corneal epithelium's ability to remodel in response to changes in stromal surface curvature may have significant implications for corneal power calculations and IOL power predictions.^13^, ^14^ Recent advancements in anterior segment optical coherence tomography (AS‐OCT) have enabled comprehensive and accurate assessments of the epithelial thickness and height profile. These advancements highlight the importance of the epithelium in corneal biomechanics and refractive outcomes, allowing for the calculation of corneal power using corneal models with three refractive surfaces (i.e., anterior epithelium, stroma and posterior cornea).

This study aims to investigate the influence of the corneal epithelium and stromal curvature on corneal power calculations, particularly in cases with altered corneal anatomy such as post‐LVC and KC. By incorporating epithelial thickness and stromal curvature into these calculations, we seek to enhance the accuracy of corneal power estimations and subsequently IOL power predictions in complex corneal conditions. The findings from this study could lead to improved diagnostic and calculation methods, ultimately enhancing visual outcomes for patients undergoing cataract surgery or refractive corrections.

## PATIENTS AND METHODS

### Study design

This retrospective study conformed to ethics codes based on the tenets of the Declaration of Helsinki. Prior ethics approval was obtained (Ärztekammer des Saarlandes, 157/21). The eyes of four cataract surgery patients showing exemplary cases (normal cornea, after myopic LVC, after hyperopic LVC and with KC) are presented.

### Data export

Measurement data were used from a high‐resolution anterior segment analyser (MS‐39, CSO, csoitalia.it/), exported with the standard user software to a CSV data file. All names and dates of birth were removed to ensure patient anonymity. The data file consists of corneal curvature and power data, as well as surface height data (epithelium, stroma and endothelium), organised in a cylindrical coordinate system. This system includes 256 equidistant semi‐meridians and 30 radial distances, ranging from 0.2 to 6 mm from the centre in 0.2 mm increments. For analysis of the spherical fit, all 256 × 30 data points within the 12‐mm zone were evaluated. Each data block comprises 256 azimuthal data points with a radial spacing from 0.2 to 6 mm, incremented by 0.2 mm. A floating best fit sphere was fitted to all three surfaces within the central 2.0, 3.2, 4.0, 5.2 and 6.0 mm zones in terms of minimising the root‐mean‐squared fit error to read out the mean radii of curvature R1, R2 and R3 and the centres of the spheres. The apices were extracted from the centres and the radii of curvature, and the radii of curvature and apices were used for the paraxial calculations.

### Exemplary cases

Four exemplary cases are presented (normal cornea, after myopic LVC, after hyperopic LVC and KC) to show how the interplay of epithelial and stromal characteristics influences corneal power. To achieve this objective, each cornea is subjected to calculations considering one, two or three refractive surfaces.

### Parameters used for calculation

For the refractive indices, we use 1.0 for air and literature data for the epithelium, stroma and aqueous humour.^15^, ^16^, ^17^, ^18^ Parameters considered in the calculations are:

1. Refractive index of air (*n*_1_ = 1.0).
2. Refractive index of the epithelium (*n*_2_ = 1.40).
3. Refractive index of the stroma/cornea (*n*_3_ = 1.376).
4. Refractive index of the aqueous (*n*_4_ = 1.336).
5. Keratometric index (If referenced to the back surface vertex plane [BV], power *n*_K_ = 1.3375; if referenced to the front surface vertex plane [FV], power *n*_K_ = 1.332; if referenced to the image‐sided principal plane [IPP], power *n*_K_ = 1.3315; see Table S1).
6. Epithelial front radius of curvature (radius *R*_1f_ in the flattest and *R*_1s_ in the steepest meridian).
7. Stromal front radius of curvature (radius *R*_2f_ in the flattest and *R*_2s_ in the steepest meridian).
8. Corneal back surface radius of curvature (radius *R*_3f_ in the flattest and *R*_3s_ in the steepest meridian).
9. Central epithelial thickness (*T*_1_).
10. Central stromal thickness (*T*_2_).
11. Central total corneal thickness (*T*_1+2_).
12. Corneal power (*F*_C_).
13. Power of the epithelial front surface (*F*_1_).
14. Power of the stromal front surface (*F*_2_).
15. Power of the corneal back surface (*F*_3_).

### Calculation of corneal power

**FIGURE 1:**
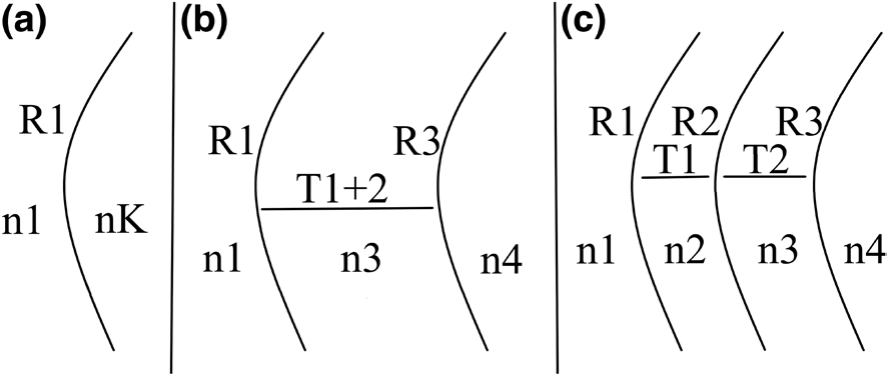
Schematics of a (a) one, (b) two and (c) three refractive surfaces model cornea. *n*1, Refractive index of air; *n*2, refractive index of epithelium; *n*3, refractive index of stroma/corneal tissue; *n*4, refractive index of aqueous; *n*_K_, keratometer index; *R*_1_, epithelial front radius of curvature; *R*_2_, stromal front radius of curvature; *R*_3_, corneal back surface radius of curvature.

These calculations are based on three different model corneas. Model one, two and three considers one, two or three refractive surfaces, respectively (Figure 1).

As a simplification, harmonic mean calculation of epithelial front surface radius of curvature, stromal front surface radius of curvature and corneal back surface radius of curvature are used in the processing:

$$R_{1}=\frac{2\times R_{1f}\times R_{1s}}{R_{1f}+R_{1s}}$$

$$R_{2}=\frac{2\times R_{2f}\times R_{2s}}{R_{2f}+R_{2s}}$$

$$R_{3}=\frac{2\times R_{3f}\times R_{3s}}{R_{3f}+R_{3s}}$$

Unlike the arithmetic mean, the harmonic mean refers to the spherical equivalent power of the spherocylindrical corneal surface. With corneal astigmatism, the arithmetic mean always overestimates corneal radius of curvature and underestimates corneal power. The difference between the harmonic and the arithmetic mean increases with the difference between the steep and flat radii of curvature, which is especially important for irregular or ectatic corneas.

A three component system, as described by Smith and Atchison,^19^ can be used. In corneal power calculations, the focal length or power can be referenced to the image sided principal plane, to the front surface vertex plane or to the back surface vertex plane. Usually, a keratometer index of 1.332 is used with IPP power, a keratometer index of 1.3315 is used with FV power and 1.3375 is used with BV power.^20^ Comparisons of corneal power calculations including one, two or three refractive surfaces should all be referenced to the same plane. The principal plane position depends on the selected corneal model. This is not an issue if power is referenced to the front or back surface.

These calculations are referenced to the IPP results as follows:

1. A simplified one refractive surface approach (Figure 1a).
$$F_{c1}=\left(\frac{n_{k}-n_{1}}{R_{1}}\right)$$

where $n_{k}=1.3315$.

2 A two refractive surfaces approach (Figure 1b):
$$F_{c2}=F_{1}+F_{3}-\left(\frac{T_{1+2}}{n_{3}}\right)\times F_{1}\times F_{3}$$

where $F_{1}=\left(\frac{n_{3}-n_{1}}{R_{1}}\right)$, $F_{3}=\left(\frac{n_{4}-n_{3}}{R_{3}}\right)$.

3 A three refractive surfaces approach (Figure 1c):
$$F_{c3}=F_{1}+F_{2}+F_{3}-\frac{T_{1}\times F_{1}\times F_{2}}{n_{2}}-\frac{T_{2}\times F_{2}\times F_{3}}{n_{3}}-\left(\frac{T_{1}}{n_{2}}+\frac{T_{2}}{n_{3}}\right)\times F_{1}\times F_{3}+\frac{T_{1}\times T_{2}\times F_{1}\times F_{2}\times F_{3}}{n_{2}\times n_{3}}$$

where $F_{1}=\left(\frac{n_{2}-n_{1}}{R_{1}}\right)$, $F_{2}=\left(\frac{n_{3}-n_{2}}{R_{2}}\right),$ $F_{3}=\left(\frac{n_{4}-n_{3}}{R_{3.}}\right)$.

Corneal front vertex power is obtained according to the followed equations:

4 A simplified one refractive surface approach.
$$F_{c4}=\left(\frac{n_{k}-n_{1}}{R_{1}}\right)$$

where $n_{k}=1.332$.

5 A two refractive surfaces approach:
$$F_{c5}=\frac{F_{1}+F_{3}-\frac{T_{1+2}}{n_{3}}\times F_{1}\times F_{3}}{1-\frac{T_{1+2}}{n_{3}}\times F_{3}}$$

where $F_{1}=\left(\frac{n_{3}-n_{1}}{R_{1}}\right)$, $F_{3}=\left(\frac{n_{4}-n_{3}}{R_{3}}\right)$.

6 A three refractive surfaces approach:
$$F_{c6}=\frac{F_{1}+F_{2}+F_{3}-\frac{T_{1}}{n_{1}}\times F_{1}\times \left(F_{2}+F_{3}\right)-\frac{T_{2}}{n_{2}}\times F_{3}\times \left(F_{1}+F_{2}\right)+\frac{T_{1}\times T_{2}}{n_{1}\times n_{2}\times F_{1}\times F_{2}\times F_{3}}\;}{1-\frac{T_{1}}{n_{1}}\times \left(F_{2}+F_{3}\right)-\frac{T_{2}}{n_{2}}\times F_{3}+\frac{T_{1}\times T_{2}}{n_{1}\times n_{2}\times F_{2}\times F_{3}}\;}$$

where $F_{1}=\left(\frac{n_{2}-n_{1}}{R_{1}}\right),$ $F_{2}=\left(\frac{n_{3}-n_{2}}{R_{2}}\right),$ $F_{3}=\left(\frac{n_{4}-n_{3}}{R_{3}}\right).$

Instead of referencing powers to the FV, they can be referenced to the back surface, giving BV powers as follows:

7 A simplified one refractive surface approach.
$$F_{c7}=\left(\frac{n_{k}-n_{1}}{R_{1}}\right)$$

where $n_{k}=1.3375$.

8 A two refractive surfaces approach:
$$F_{c8}=\frac{F_{1}+F_{3}-F_{1}\times F_{3}\times \frac{T_{1+2}}{n_{3}}}{1-F_{1}\times \frac{T_{1+2}}{n_{3}}}$$

where $F_{1}=\left(\frac{n_{3}-n_{1}}{R_{1}}\right)$, $F_{3}=\left(\frac{n_{4}-n_{3}}{R_{3}}\right).$

9 A three refractive surfaces approach:
$$F_{c9}=\frac{F_{1}+F_{2}+F_{3}-\frac{T_{2}}{n_{3}}\times F_{3}\times \left(F_{1}+F_{2}\right)-\frac{T_{1}}{n_{2}}\times F_{1}\times \left(F_{2}+F_{3}\right)+\frac{T_{1}\times T_{2}}{n_{2}\times n_{3}\times F_{1}\times F_{2}\times F_{3}}}{1-\frac{T_{2}}{n_{3}}\times \left(F_{1}+F_{2}\right)-\frac{T_{1}}{n_{2}}\times F_{1}+\frac{T_{1}\times T_{2}}{n_{2}\times n_{3}\times F_{1}\times F_{3}}}$$

where $F_{1}=\left(\frac{n_{2}-n_{1}}{R_{1}}\right),$ $F_{2}=\left(\frac{n_{3}-n_{2}}{R_{2}}\right),$ $F_{3}=\left(\frac{n_{4}-n_{3}}{R_{3}}\right).$

### Statistical analysis

Data were analysed using Microsoft Excel (v.16.12, Microsoft.com). Descriptive statistics are provided in the tables. Differences between the corneal powers of the two and three surface models are calculated by subtracting the corneal power of the two surface models from the three surface models.

## RESULTS

### Normal cornea

**FIGURE 2:**
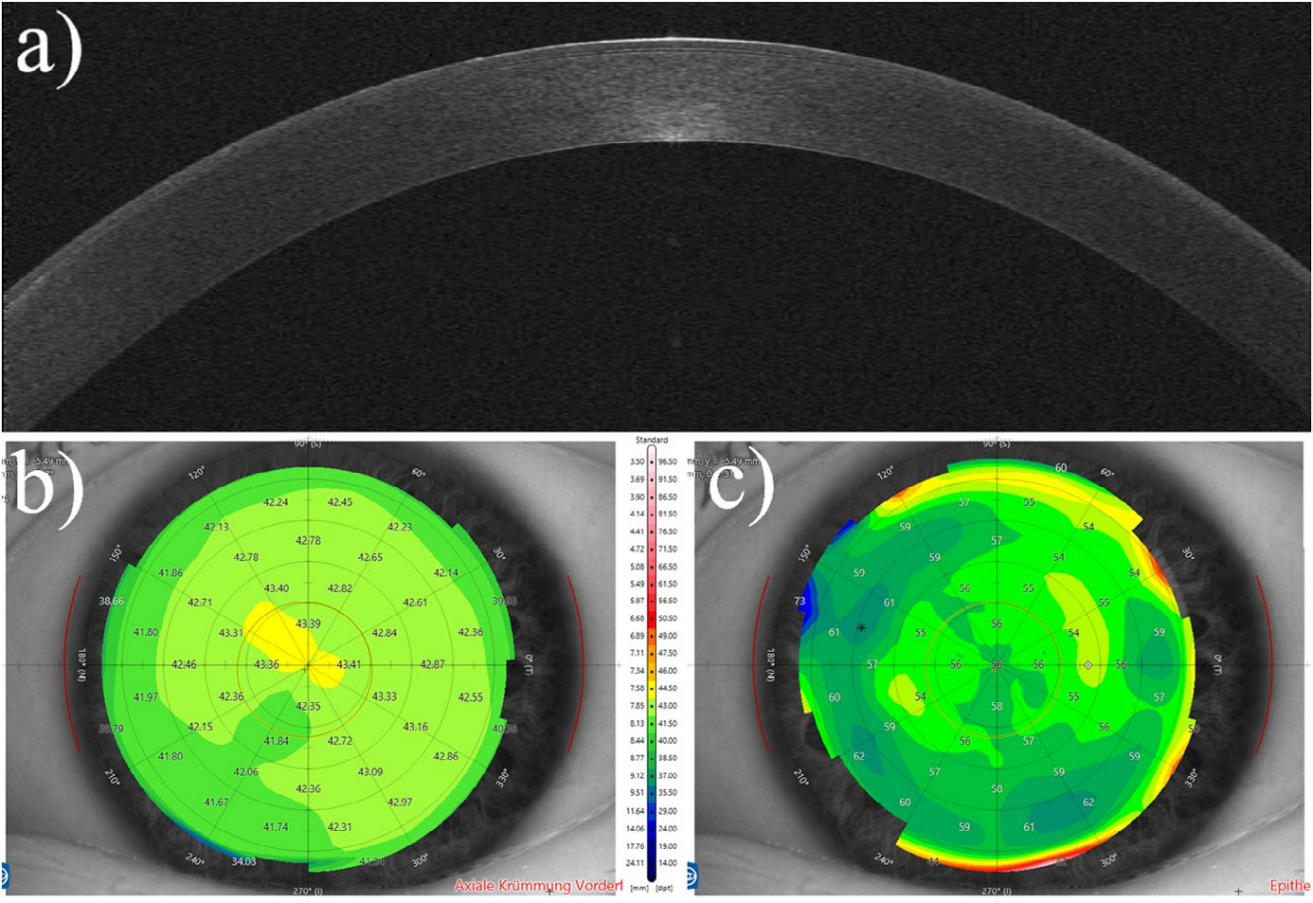
A normal cornea with normal stromal and epithelial thicknesses. (a) The optical coherence tomography scan. (b) The axial curvature map, also known as a sagittal map. (c) Epithelial thickness mapping.

Exemplary case number is an untreated, healthy cornea (Figure 2).

**FIGURE 3:**
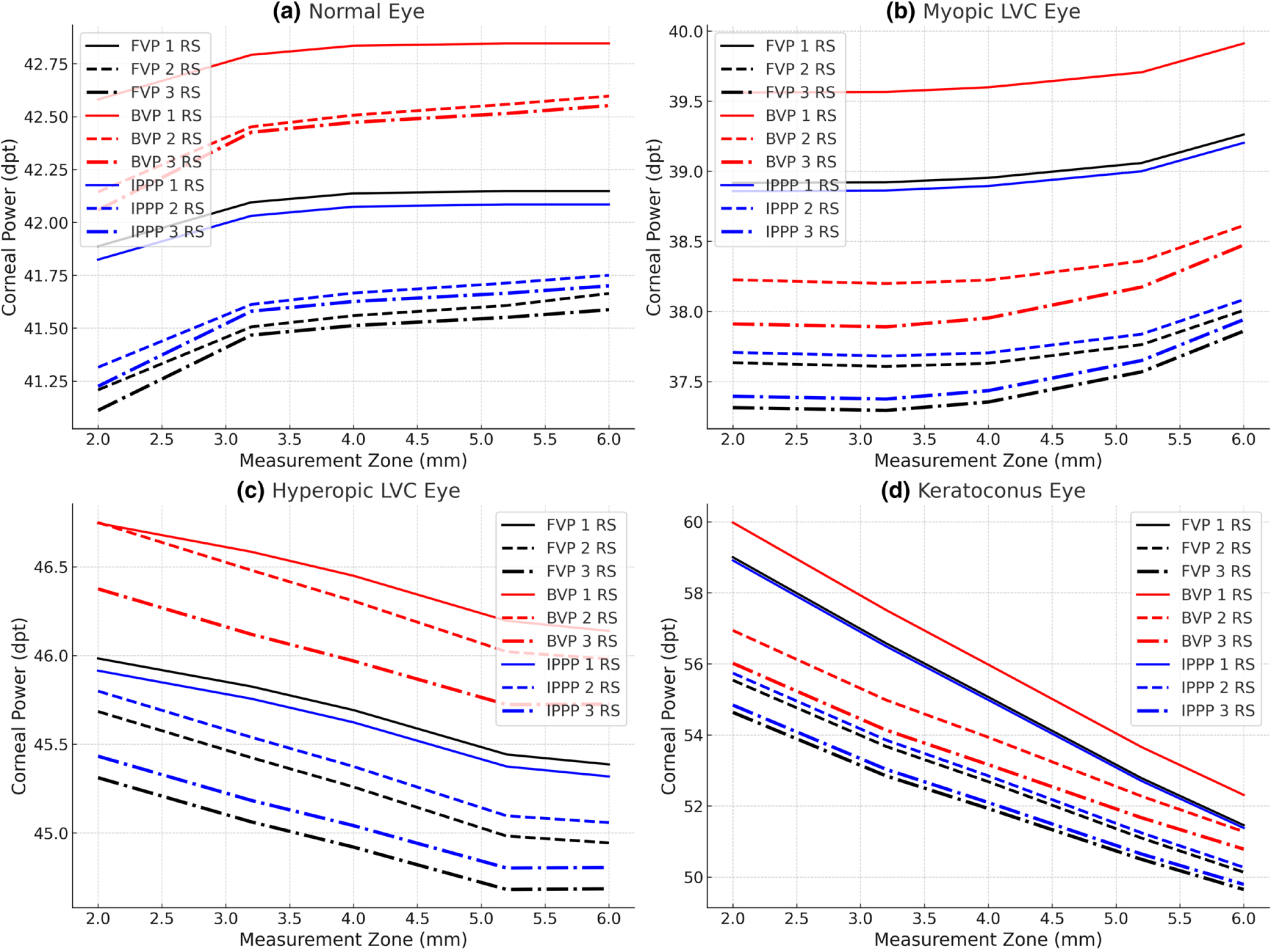
Corneal powers across the measurement zones for the three models (1 refractive surfaces [RS], 2 RS and 3 RS). Black lines represent front vertex power (FVP), red lines represent back vertex power (BVP) and blue lines represent image sided principal plane power (IPPP). (a) A normal cornea; (b) a cornea after myopic laser vision correction (LVC); (c) a cornea after hyperopic LVC and (d) a cornea with keratoconus.

Table 1 shows corneal radii of curvature and corneal thicknesses for a range of measurement zones, while Table 2 and Figure 3a have IPP power, FV power and BV powers for each combination of surface number and measurement zone.

**TABLE 1:** Normal eye. Anterior corneal radius of curvature (*R*_1_), anterior stromal radius of curvature (*R*_2_), posterior corneal radius of curvature (*R*_3_), epithelial thickness (*T*_1_), stromal thickness (*T*_2_) and total corneal thickness (*T*_1+2_) for a 2.0, 3.2, 4.0, 5.2 and 6.0 mm measurement zone.

	*R*_1_ (mm)	*R*_2_ (mm)	*R*_3_ (mm)	*T*_1_ (mm)	*T*_2_ (mm)	*T*_1+2_ (mm)
2.0 mm	7.926	7.782	6.403	0.057	0.514	0.570
3.2 mm	7.887	7.824	6.469	0.057	0.514	0.571
4.0 mm	7.879	7.805	6.474	0.057	0.514	0.571
5.2 mm	7.877	7.791	6.512	0.057	0.515	0.571
6.0 mm	7.877	7.789	6.552	0.057	0.516	0.572

**TABLE 2:** Normal eye. Front vertex power (FVP), back vertex power (BVP) and image‐sided principal plane power (IPPP) in dioptres for one refractive surface (1 RS), two refractive surfaces (2 RS) and three refractive surfaces (3 RS) models in various measurement zones.

Measurement zone (mm)	FVP			BVP			IPPP		
	1 RS	2 RS	3 RS	1 RS	2 RS	3 RS	1 RS	2 RS	3 RS
2.0	41.887	41.208	41.111	42.581	42.144	42.058	41.824	41.315	41.225
3.2	42.095	41.506	41.467	42.792	42.452	42.426	42.031	41.612	41.580
4.0	42.137	41.559	41.512	42.835	42.507	42.473	42.074	41.666	41.626
5.2	42.148	41.607	41.551	42.846	42.558	42.515	42.085	41.713	41.665
6.0	42.148	41.664	41.587	42.846	42.597	42.552	42.085	41.750	41.700

The effect of the epithelium is given by subtracting the powers of two surface models from the corresponding powers of the three surface models. Table 3 shows these results for FV power. The effect of the epithelium on FV power is between −0.03 and −0.10 D and decreases with increase in the size of the measurement zone. Additionally, Table 4 displays absolute differences between the one refractive surface model and the two and three refractive surfaces models.

**TABLE 3:** Differences in corneal front vertex power (FVP) between the two and three refractive surfaces models in dioptres for each measurement zone. KC, keratoconus; LVC, laser vision correction.

Difference in FVP (mm)	Normal	Myopic LVC	Hyperopic LVC	KC
2.0	−0.097	−0.319	−0.373	−0.905
3.2	−0.039	−0.313	−0.362	−0.830
4.0	−0.047	−0.276	−0.339	−0.756
5.2	−0.056	−0.194	−0.301	−0.599
6.0	−0.057	−0.148	−0.259	−0.487

**TABLE 4:** Absolute differences in corneal front vertex power (FVP) between the one, two and three refractive surfaces models for the various measurement zones. The comparisons are marked as 3v2 for the three and two refractive surfaces models, as 3v1 for the three and one refractive surfaces models and 2v1 for the two and one refractive surfaces models. KC, keratoconus; LVC, laser vision correction.

Difference in FVP (D) (mm)	Normal eye			Myopic LVC			Hyperopic LVC			KC		
	3v2	3v1	2v1	3v2	3v1	2v1	3v2	3v1	2v1	3v2	3v1	2v1
2.0	0.10	0.78	0.68	0.32	1.60	1.28	0.37	0.67	0.30	0.91	4.37	3.46
3.2	0.04	0.63	0.59	0.31	1.63	1.31	0.36	0.76	0.40	0.83	3.73	2.90
4.0	0.05	0.63	0.58	0.28	1.60	1.32	0.34	0.77	0.43	0.76	3.14	2.39
5.2	0.06	0.60	0.54	0.19	1.49	1.30	0.30	0.76	0.46	0.60	2.29	1.69
6.0	0.08	0.56	0.48	0.15	1.40	1.25	0.26	0.70	0.44	0.49	1.81	1.32

### Myopic LVC

**FIGURE 4:**
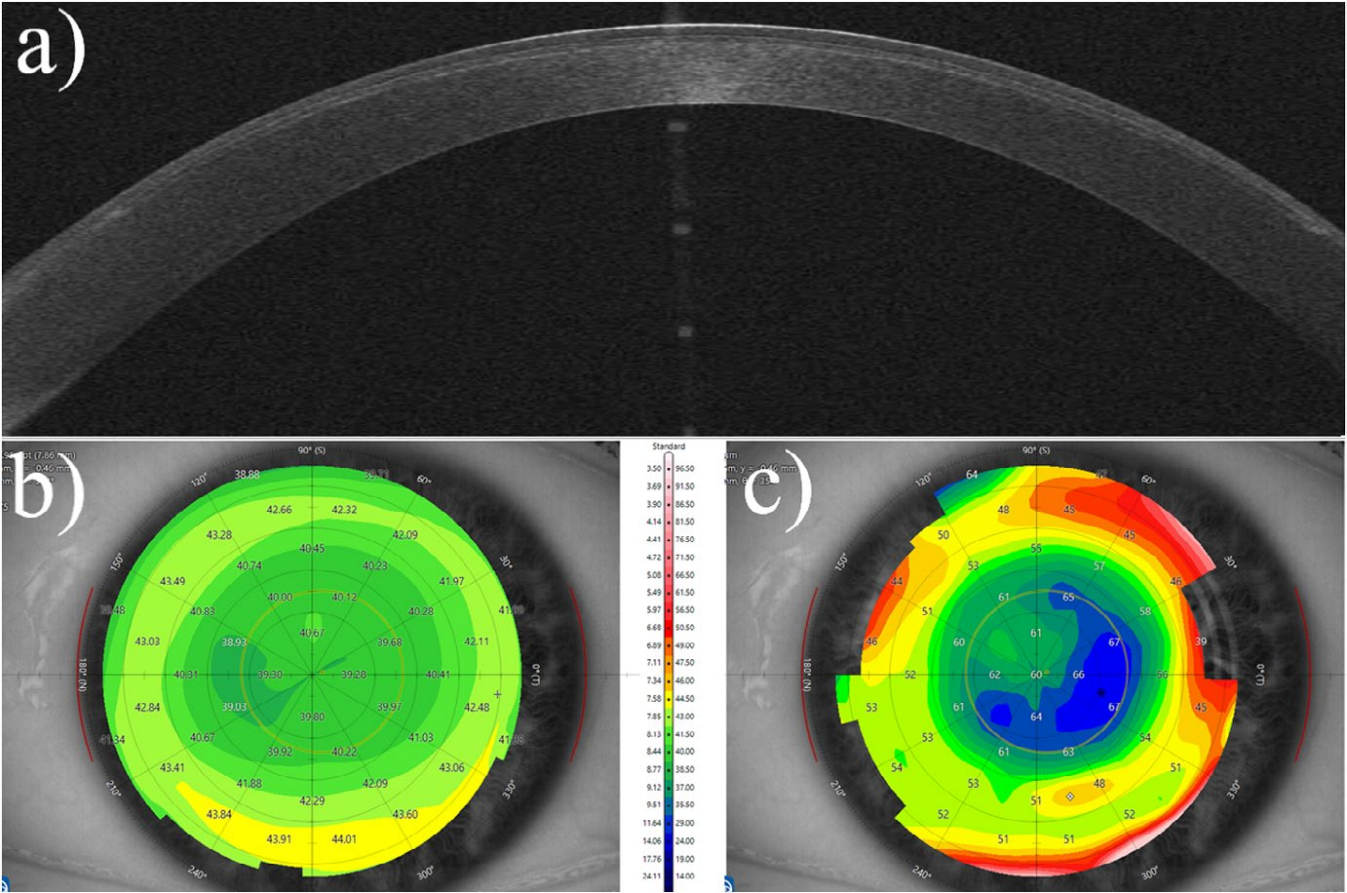
A post‐myopic laser vision correction (LVC) cornea with a centrally thin stroma characterised by a flat anterior surface. Atop this altered stromal configuration, there is a compensatory mechanism evidenced by a thickening of the epithelium. (a) The optical coherence tomography scan; (b) the axial curvature map, also known as a sagittal map. (c) Epithelial thickness mapping.

Exemplary case number two shows the cornea of an eye after myopic LVC (Figure 4).

Table 5 shows corneal radii of curvature and corneal thicknesses for a range of measurement zones, and Table 6 and Figure 3b have IPP power, FV power and BV powers for each combination of surface number and measurement zone.

**TABLE 5:** Myopic laser vision correction eye. Anterior corneal radius of curvature (*R*_1_), anterior stromal radius of curvature (*R*_2_), posterior corneal radius of curvature (*R*_3_), epithelial thickness (*T*_1_), stromal thickness (*T*_2_) and total corneal thickness (*T*_1+2_) for each measurement zone.

Measurement zone (mm)	*R*_1_ (mm)	*R*_2_ (mm)	*R*_3_ (mm)	*T*_1_ (mm)	*T*_2_ (mm)	*T*_1+2_ (mm)
2.0	8.531	8.024	6.198	0.061	0.362	0.423
3.2	8.530	8.032	6.168	0.061	0.361	0.423
4.0	8.523	8.080	6.155	0.062	0.361	0.423
5.2	8.500	8.183	6.168	0.063	0.360	0.423
6.0	8.456	8.212	6.182	0.064	0.360	0.424

**TABLE 6:** Myopic laser vision correction eye. Front vertex power (FVP), back vertex power (BVP) and image sided principal plane power (IPPP) in dioptres for one refractive surface (1 RS), two refractive surfaces (2 RS) and three refractive surfaces (3 RS) models in various measurement zones.

Measurement zone (mm)	FVP			BVP			IPPP		
	1 RS	2 RS	3 RS	1 RS	2 RS	3 RS	1 RS	2 RS	3 RS
2.0	38.917	37.634	37.314	39.562	38.226	37.911	38.858	37.708	37.395
3.2	38.921	37.608	37.294	39.566	38.199	37.890	38.863	37.682	37.375
4.0	38.953	37.630	37.354	39.599	38.224	37.953	38.895	37.705	37.435
5.2	39.059	37.763	37.569	39.706	38.360	38.174	39.000	37.838	37.650
6.0	39.262	38.008	37.860	39.912	38.613	38.474	39.203	38.084	37.942

Tables 3 and 6 show that the effect of the epithelium on FV power is between −0.14 and −0.32 D and decreases with increase in the size of the measurement zone.

### Hyperopic LVC

**FIGURE 5:**
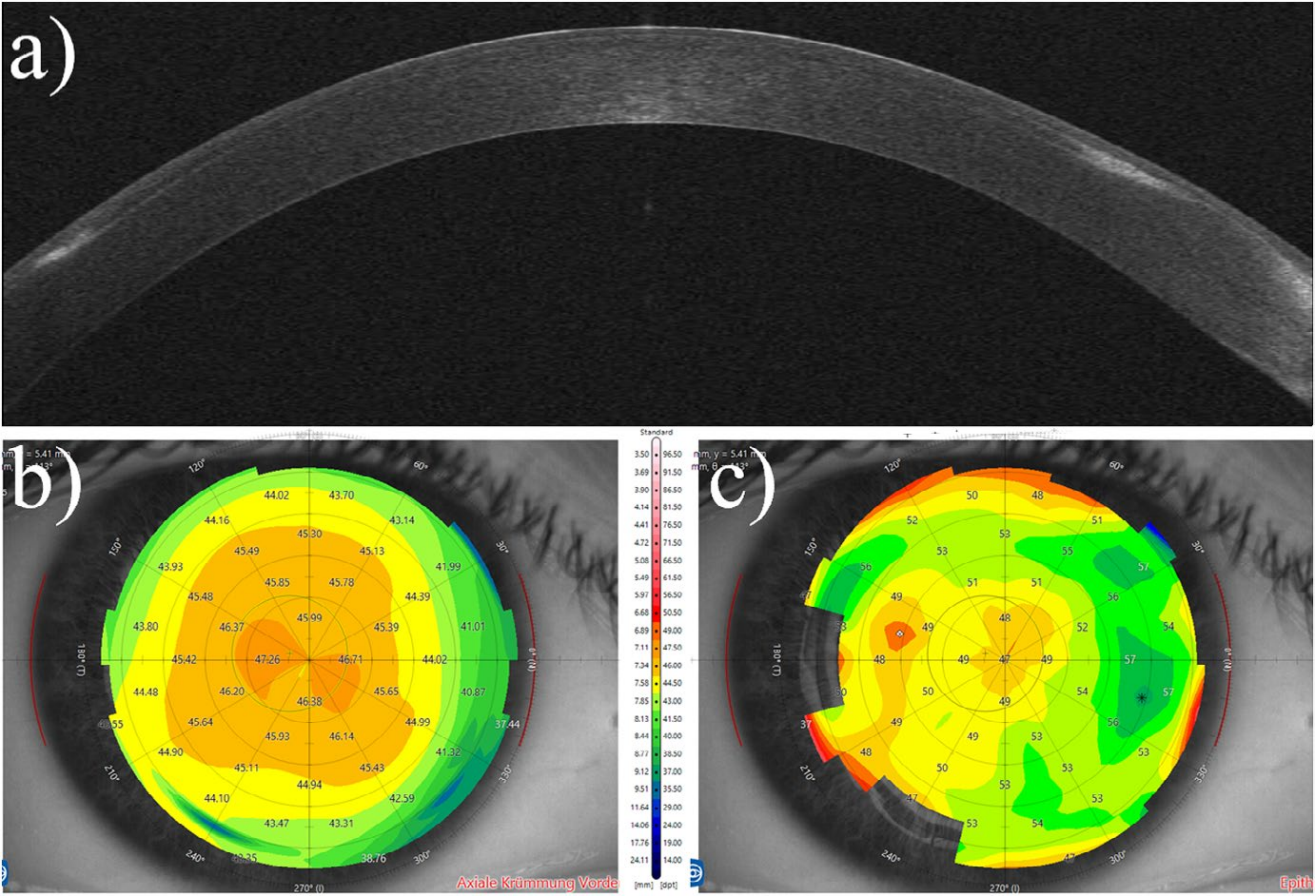
A post‐hyperopic laser vision correction cornea showing a normal central thickness, diminished peripheral stromal thickness and a flattish anterior surface. Notably, the epithelium maintains its naturally flat anterior surface, resulting in central thinning, but an increase in peripheral epithelial thickness as a compensatory response. (a) The optical coherence tomography scan; (b) the axial curvature map, also known as a sagittal map. (c) Epithelial thickness mapping.

Exemplary case number three is on the cornea of an eye after hyperopic LVC (Figure 5).

Table 7 shows corneal radii of curvature and corneal thicknesses for a range of measurement zones, and Table 8 and Figure 3c have powers for each combination of surface number and measurement zone.

**TABLE 7:** Hyperopic laser vision correction eye. Anterior corneal radius of curvature (*R*_1_), anterior stromal radius of curvature (*R*_2_), posterior corneal radius of curvature (*R*_3_), epithelial thickness (*T*_1_), stromal thickness (*T*_2_) and total corneal thickness (*T*_1+2_) for various measurement zones.

Measurement zone (mm)	*R*_1_ (mm)	*R*_2_ (mm)	*R*_3_ (mm)	*T*_1_ (mm)	*T*_2_ (mm)	*T*_1+2_ (mm)
2.0	7.220	6.797	6.241	0.047	0.490	0.538
3.2	7.245	6.831	6.163	0.047	0.490	0.537
4.0	7.266	6.874	6.149	0.047	0.489	0.537
5.2	7.306	6.952	6.155	0.048	0.489	0.537
6.0	7.315	7.006	6.179	0.049	0.488	0.537

**TABLE 8:** Hyperopic laser vision correction eye. Front vertex power (FVP), back vertex power (BVP) and image sided principal plane power (IPPP) in dioptres for one refractive surface (1 RS), two refractive surfaces (2 RS) and three refractive surfaces (3 RS) models in various measurement zones.

Measurement zone (mm)	FVP			BVP			IPPP		
	1 RS	2 RS	3 RS	1 RS	2 RS	3 RS	1 RS	2 RS	3 RS
2.0	45.983	45.684	45.311	46.745	46.749	46.375	45.914	45.799	45.432
3.2	45.825	45.424	45.062	46.584	46.480	46.119	45.756	45.539	45.183
4.0	45.692	45.259	44.920	46.449	46.307	45.969	45.623	45.374	45.041
5.2	45.442	44.982	44.681	46.195	46.021	45.723	45.374	45.096	44.802
6.0	45.386	44.944	44.685	46.138	45.980	45.725	45.318	45.058	44.805

Tables 3 and 8 show that the effect of the epithelium on FV power is between −0.25 and −0.37 D and decreases with increase in the size of the measurement zone.

### Keratoconus

**FIGURE 6:**
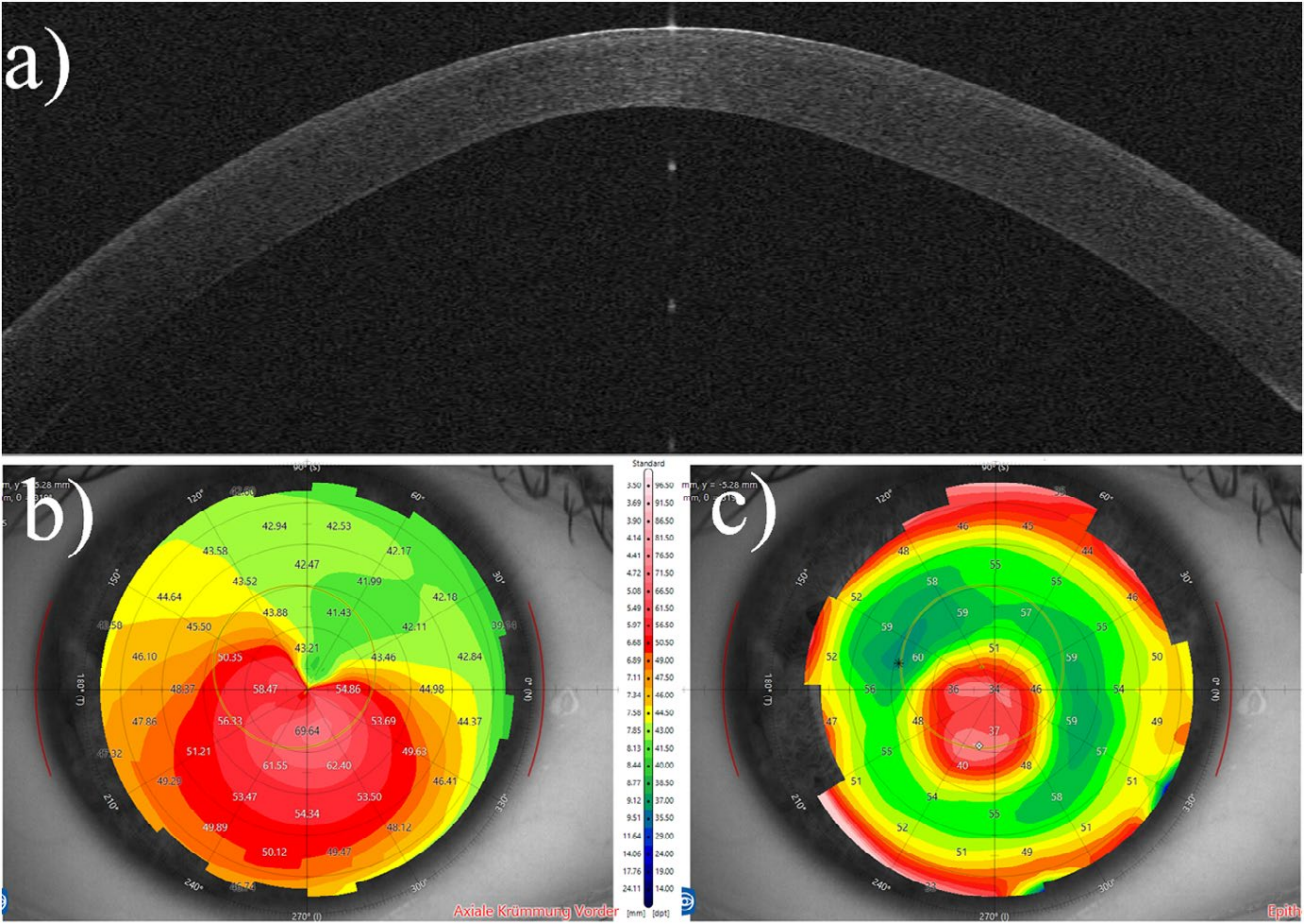
A cornea affected by keratoconus, with a distinctive pattern characterised by a steep inferior cornea with decentred epithelial thinning (usually found at the location of the thinnest corneal/stromal thickness). (a) The optical coherence tomography scan; (b) the axial curvature map, also known as a sagittal map. (c) Epithelial thickness mapping.

Exemplary case number four is on the cornea of an untreated eye with keratoconus (Figure 6).

Table 9 shows corneal radii of curvature and corneal thicknesses for a range of measurement zones, and Table 10 and Figure 3d have powers for each combination of surface number and measurement zone.

**TABLE 9:** Keratoconic eye. Anterior corneal radius of curvature (*R*_1_), anterior stromal radius of curvature (*R*_2_), posterior corneal radius of curvature (*R*_3_), epithelial thickness (*T*_1_), stromal thickness (*T*_2_) and total corneal thickness (*T*_1+2_) for various measurement zones.

Measurement zone (mm)	*R*_1_ (mm)	*R*_2_ (mm)	*R*_3_ (mm)	*T*_1_ (mm)	*T*_2_ (mm)	*T*_1+2_ (mm)
2.0	5.627	5.037	3.533	0.038	0.397	0.435
3.2	5.868	5.277	3.833	0.038	0.402	0.440
4.0	6.029	5.457	4.118	0.038	0.406	0.444
5.2	6.290	5.788	4.594	0.040	0.414	0.454
6.0	6.452	6.016	4.899	0.042	0.420	0.462

**TABLE 10:** Keratoconic eye. Front vertex power (FVP), back vertex power (BVP) and image sided principal plane power (IPPP) in dioptres for one refractive surface (1 RS), two refractive surfaces (2 RS) and three refractive surfaces (3 RS) models in various measurement zones.

Measurement zone (mm)	FVP			BVP			IPPP		
	1 RS	2 RS	3 RS	1 RS	2 RS	3 RS	1 RS	2 RS	3 RS
2.0	59.001	55.539	54.634	59.979	56.941	56.016	58.912	55.738	54.838
3.2	56.578	53.675	52.845	57.515	54.981	54.134	56.493	53.854	53.030
4.0	55.067	52.682	51.926	55.979	53.933	53.162	54.984	52.847	52.097
5.2	52.782	51.095	50.496	53.657	52.273	51.667	52.703	51.242	50.646
6.0	51.457	50.134	49.647	52.309	51.275	50.785	51.379	50.271	49.791

Tables 3 and 10 show that the effect of the epithelium on front vertex power is between −0.48 and −0.93 D and decreases with increase in the size of the measurement zone.

Table 4 shows the absolute differences in FV powers between the model eyes with different numbers of surfaces. The differences between the one refractive surface model and the other models were larger than the differences between the two and three refractive surfaces models, with the exception of the 2.0 mm zone of the cornea with hyperopic LVC. The differences between one or more refractive surfaces were largest in the exemplary corneas with previous LVC and KC.

## DISCUSSION

Historically, keratometers and biometers have measured the anterior corneal surface and empirically factored in corneal thickness and the posterior curvature to determine corneal power. With the availability of devices that measure the posterior surface, the posterior curvature of the cornea can be used, especially in the management of astigmatism.^7^, ^8^ The current generation of OCT tomography devices allows the inclusion of measurements of the epithelium and stroma such as epithelial thickness and epithelial and stromal height map data. Here, epithelial and stromal data were used to calculate the stromal anterior curvature and determine the effect of the epithelium on corneal power, showing how to incorporate the epithelium into corneal power calculations. Analysing epithelial power alone (as provided by the device) will not be sufficient to understand its effect on corneal power; rather, a combination of epithelial and stromal power measurements is necessary to address this question comprehensively.

In refractive surgery, the epithelium has been notorious for posing a problem in hyperopic corrections, where corneal remodelling often leads to regression and irregular astigmatism.^21^, ^22^, ^23^, ^24^ However, the influence of the epithelium and stromal curvature on corneal power extends to other cases. Four cases were examined to explore the impact of including stromal curvature and epithelial thickness, quantified by the difference between the models incorporating two and three refractive surfaces. Traditional keratometry measures corneal curvature in a very small central zone (~2.5 mm), assuming a spherical surface. Typically, modern biometers measure curvature across a larger zone (~3–4 mm), incorporating more central corneal data. The current study, however, fits a spherical model for various diameters/zones, without accounting for asphericity, which is often modelled using conic sections. These methodological differences highlight the need for careful interpretation when comparing results across different instruments and studies. In these examples, the greatest effect was observed in the case of KC, where the difference was 0.83 D for a 3.2‐mm measurement zone (Table 3). This was followed by hyperopic LVC where corneal power decreased by 0.36 D in a 3.2‐mm zone, and myopic LVC where corneal power decreased by 0.31 D in a 3.2‐mm zone. By contrast, the normal cornea showed a corresponding decrease of only 0.04 D. It is important to note that these effects are case‐specific and will vary with the severity of the disease or surgical changes. Naturally, the principal plane is located very close to the anterior corneal surface. As a result, the differences in corneal power when referenced to the IPP plane and FV plane were smaller than when either were referenced to the BV plane.

In all four exemplary cases, the influence of the epithelium increased as the measurement zone (a measurement for a particular distance away from the centre) became smaller. Nonetheless, asphericity, astigmatism and further surface fit models are not considered in this study and will be the subject of further investigations. These findings underscore the importance of thorough preoperative diagnostics, particularly concerning photopic and mesopic pupil sizes, as well as the functional optical zone, especially in special cases. The influence of the epithelium on corneal power, and consequently on the ideal IOL power, can vary depending on the individual pupil size. It is important to note, however, that the impact of aligning pupil size with the keratometry measurement zone on refraction has not been proven and remains to be explored.^25^ The effect of the epithelium on corneal power highlights the importance of developing or utilising a formula that does not rely on corneal power in the effective lens position algorithm for complex cases, such as in KC or post‐LVC eyes. While empirical elements in a formula can partially address this issue, the standard deviation remains higher compared to that in a normal population. This indicates a need for further refinement and adjustment of these formulas to improve accuracy and outcomes in these special cases.^9^, ^10^

Given the reduced refractive accuracy in KC or post‐LVC eyes during cataract surgery, adaptations in surgical planning are necessary. In larger clinics, these cases might be overlooked if pre‐examinations are conducted by fluctuating personnel rather than the surgeon themselves. Currently, the anterior to posterior corneal curvature ratio can identify eyes with pathologies or those that have undergone pre‐surgical interventions such as LVC.^26^ Fixed anterior to posterior corneal curvature ratios are also useful for thick lens corneal power calculations.^27^, ^28^ In the future, the anterior corneal to anterior stromal curvature ratio or anterior stromal curvature to posterior curvature ratio may further improve detection algorithms. These ratios could contribute to three refractive surface model corneal power calculations. Establishing normative values and understanding their distributions will be the focus of upcoming studies.

The difference between corneal power calculations derived from single refractive surface models using current keratometer indices (based on the Gullstrand model eye) and those derived from two or three refractive surfaces models was considerable clinically. Even in a normal cornea, the difference compared to the two refractive surfaces model ranged from 0.48 to 0.68 D, while the difference from the three refractive surfaces model ranged from 0.56 to 0.78 D. This discrepancy was more pronounced in the exemplary case of KC, where the difference, compared to the two refractive surfaces model, ranged from 1.32 to 3.46 D, and the difference to the three refractive surfaces model ranged from 1.81 to 4.37 D. These findings underscore the need to update the way corneal power distributions are displayed in tomography and keratometry devices. The next step is to analyse the influence of the epithelium and stroma on raytracing calculations of corneal power.

The present work raises several critical points and has some limitations. The internal processing strategy is unclear regarding whether epithelial thickness is measured coaxially or along the ray bundle. If the measurements are not taken along the optical path, then ray tracing through the layers would be necessary, which is more complex. The thickness values of the epithelium depend on the refractive index chosen by the device manufacturer. Typically, the refractive index selected for corneal tissue is 1.376. If this value is altered for the epithelium, it will change the estimate of angles of refraction into the cornea, which would in turn alter the epithelial thickness value and all subsequent corneal layers, assuming the thickness is measured along the ray. This creates a problem for the thickness values used in calculating powers. In the present calculations, the height data were used in the export file, which is provided in cylindrical coordinates. Since thickness is not used for the sphere fit, this problem was circumvented. However, we do not know how CSO manages the data or what simplifications are used in the software. Additionally, other anterior segment OCT devices offer epithelial thickness and height measurements but may use different display algorithms, thereby further complicating comparisons. If instruments were to provide an expanded set of parameters specifically for research purposes, potentially accompanied by a quality factor indicating the validity of the measurements, this could open avenues for more robust research. After all, the purpose of this study was not the derivation of radii of curvature from height maps but to assess the impact of epithelial and stromal curvature on corneal power. These considerations highlight the need for a standardised approach to measuring and interpreting epithelial thickness and stromal curvature, ensuring that the chosen refractive indices and models are consistent and accurately reflect the underlying anatomy. Furthermore, the refractive indices of the epithelium and stroma may be different from what was used here and may be subject to change.^16^, ^17^, ^18^

In conclusion, this is the first study to explore the impact of epithelium and stromal curvature on the total corneal power with modern AS‐OCT data. Further studies including more instruments and a larger cohort of patients in various clinical scenarios are necessary to confirm these results. In the present examples, the influence of the epithelium and stromal curvature in normal corneas was minimal, while the influence of the epithelium in corneas after myopic or hyperopic LVC and KC can be important.

## AUTHOR CONTRIBUTIONS

**Jascha A. Wendelstein:** Conceptualization (equal); data curation (equal); methodology (equal); writing – original draft (equal). **David A. Atchison:** Conceptualization (equal); methodology (equal); validation (equal); writing – review and editing (equal). **Damien Gatinel:** Resources (equal); supervision (equal); writing – review and editing (equal). **David L. Cooke:** Supervision (equal); validation (equal); writing – review and editing (equal). **Achim Langenbucher:** Conceptualization (equal); data curation (equal); formal analysis (equal); methodology (equal); resources (equal); supervision (equal); validation (equal); writing – review and editing (equal).

## FUNDING INFORMATION

The authors did not receive financial support for the research, authorship and/or publication of this article. No benefits in any form have been received or will be received from a commercial party related directly or indirectly to the subject of this article.

## CONFLICT OF INTEREST STATEMENT

Dr. Wendelstein reports research support from Carl Zeiss Meditec AG. He reports personal fees from Alcon Surgical, Bausch and Lomb, Carl Zeiss Meditec AG, Heidelberg Engineering, Rayner Surgical and Johnson & Johnson Vision outside of the submitted work. He was supported by an ‘ESCRS Peter Barry Fellowship Grant’. Dr. Gatinel reports personal fees and royalties from BVI outside the submitted work. Dr. Langenbucher reports personal fees from Hoya Surgical and Johnson & Johnson Vision outside the submitted work. There are no conflicts of interest for the other authors.

## Supporting information


Table S1.
