# Supplementary material for: The role of the epithelium in intraocular lens and corneal power calculation
Source: Ophthalmic Physiol Opt. 2025 Feb 3;45(2):372–82. doi: 10.1111/opo.13443 (PMC11823397; doi:10.1111/opo.13443)
Supplement: Supplementary file 1 — Table S1. [file OPO-45-372-s001.docx]

|  | **Supplementary Table 1:** Overview of keratometric indices and their applications in intraocular lens (IOL) power calculations |
| --- | --- |
| Keratometric indices | Keratometric index, often referred to as the Javal index or Zeiss index, can be referenced to different planes, including the front surface vertex plane, the back surface vertex plane and the image-sided principal plane. Using Gullstrand’s theoretical eye as a reference:   1. When corneal power is referenced to the image-sided principal plane, the keratometric index is 1.3315. 2. When referenced to the front vertex, the keratometric index is 1.332. 3. When referenced to the back vertex, the keratometric index is 1.337.   Historically, the value 1.337 was adjusted to 1.3375 to align a radius of curvature of 7.5 mm with a corneal power of 45.0 dioptres, ensuring compatibility with clinical keratometry measurements. |
| Keratometric indices in IOL power calculation | In IOL power calculation formulae, the spectacle back vertex distance, axial length and anterior chamber depth must be adapted to correspond with the reference point used by the keratometric index. This alignment ensures accurate representation of the optical geometry and refractive power of the cornea relative to the chosen reference plane. With the exception of the Hoffer Q formula (which uses 1.3375), all IOL calculation formulae convert the keratometric index internally to account for differences in corneal power reference points. This internal adjustment ensures that the refractive calculations remain consistent with the formula's geometric and optical assumptions. |
| Newer model eyes | The keratometric index can be adapted to newer, more anatomically accurate model eyes. These adaptations ensure compatibility with updated representations of the eye's optical properties, enabling more precise calculations in modern IOL power formulae. The need for an update to diagnostic devices to incorporate newer keratometric indices and model eyes can be a topic of further discussion. |
|  | |
